# Diagnosis and Management of Cornu Cutaneum of Nasal Vestibule: A Rare Case Report

**DOI:** 10.1155/2015/625832

**Published:** 2015-09-20

**Authors:** Hande Ezerarslan, Mustafa Mert Basaran, Sefik Halit Akmansu

**Affiliations:** Department of Otolaryngology, Ufuk University, 06520 Ankara, Turkey

## Abstract

Cornu cutaneum is a relatively uncommon projectile, irregular, hyperkeratotic nodule that can be seen in places such as scalp, forehead, eyelids, ear, nose, lips, and upper extremities which are subjectable to sunlight. Treatment is surgery with radical margins. Excisional biopsy is enough for treatment of the lesion on head and face. However, there is only little literature about cornu cutaneum on the nasal vestibule. We present an 82-year-old male patient with a necrotic, irregular shaped lesion with pedicle on the left nasal vestibule excised and diagnosed as cornu cutaneum.

## 1. Introduction

Cornu cutaneum is a relatively uncommon projectile, irregular, hyperkeratotic nodule that can be seen in places such as scalp, forehead, eyelids, ear, nose, lips, and upper extremities which are subjectable to sunlight [1]. Mostly they are believed to be benign lesions; but researches showed that they might be related to malignant or premalignant lesions [2]. It is believed that there is a relation between actinic keratosis, molluscum sebaceum, sebaceous carcinoma, warts, trichilemmoma, Bowen's disease, epidermoid carcinoma, malignant melanoma, and basal cell carcinoma and cornu cutaneum [3–5].

Cornu cutaneum is a painless, avascular, necrotic, or keratotic lesion with histopathological examination with no living cells. Certain diagnosis is always made with biopsy.

Treatment is surgery with radical margins [6]. Excisional biopsy is enough for treatment of the lesion on head and face [6]. Shaving is an option only when there is no possibility of total excision and in sensitive cosmetic areas. Electrocauterization, cryotherapy, and laser ablation are alternative methods [7]. A yearly postoperative examination should be done to control the primary malignancy and check if there are any additional malignancies.

We present an 82-year-old male patient with a necrotic lesion on the left nasal vestibule excised with excisional biopsy and diagnosed as cornu cutaneum.

## 2. Case Report

**Figure 1:**
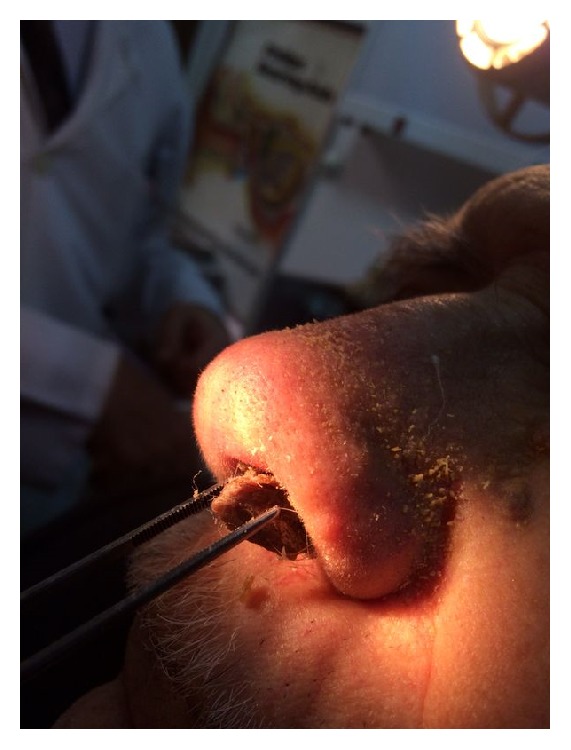
Mass was developed as seen from the left nasal cavity of lateral wall of vestibule.

**Figure 2:**
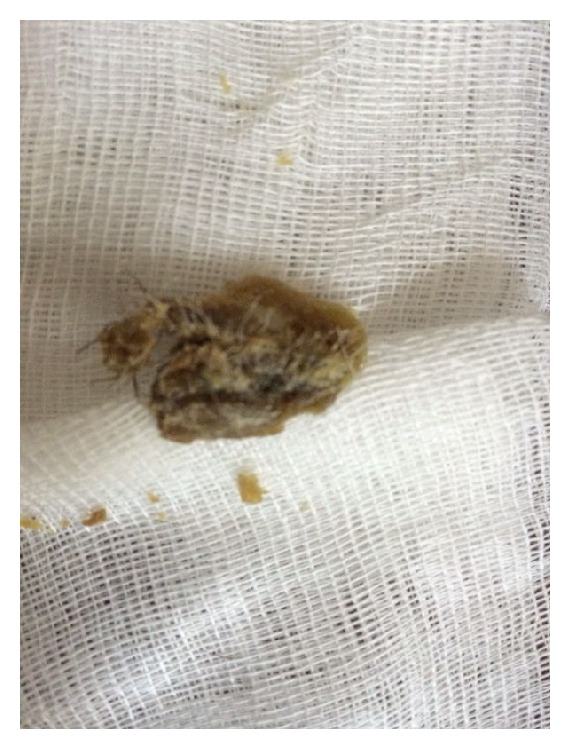
Lesion was excised by pedicle from vestibule.

**Figure 3:**
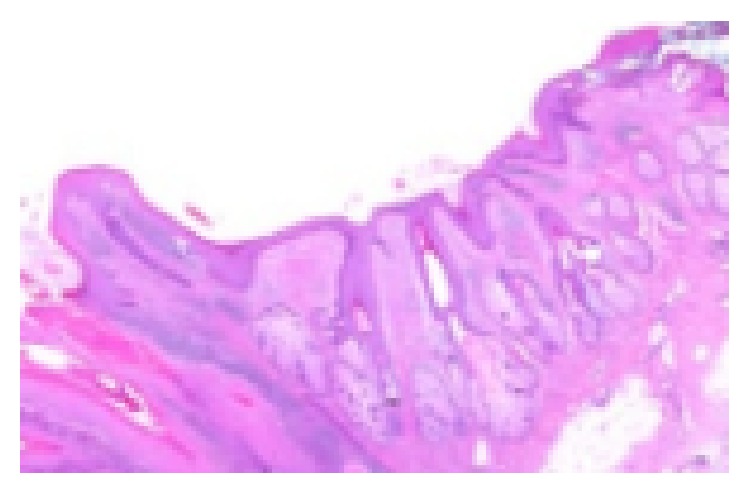
Pathological examination showed keratin lamellas which showed papillomatous improvement apart from any living cells.

**Figure 4:**
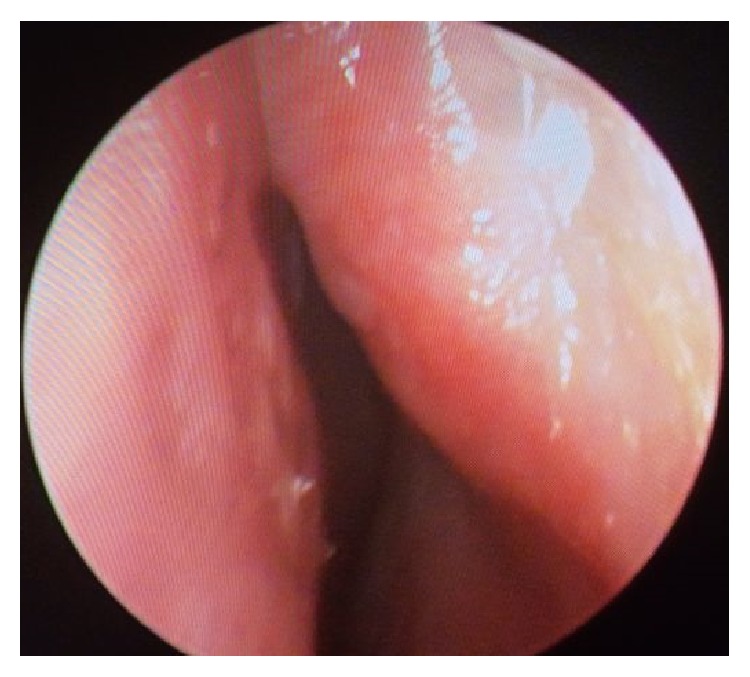
Normal physical examination was seen after 4 months postoperatively.

A male patient of 82 years of age attended our clinic with pruritus and incrustation on nasal cavity. Incrustation was increasing and, after scratching, mass has fallen to pieces without any bleeding. Mass was increased in size again in the recent month and became visible from the outside of the nose (Figure 1). Patient had hypertension and benign prostate hypertrophy. He had no history of smoking. Physical examination showed a keratotic, irregular shaped crust with a pedicle on left lateral nasal vestibule. Lesion was excised by pedicle from vestibule (Figure 2). Bleeding did not occur. Pathological examination showed keratin lamellas which showed papillomatous improvement without any living cells (Figure 3). Patient had no necrotic tissues on his vestibule after 4 months postoperatively (Figure 4).

## 3. Discussion

Cornu cutaneum is a projectile, hyperkeratotic nodule in places of the body which are sensitive to sunlight and called cutaneous horns because of their similarity of their macroscopic shape [8]. Apart from sunlight, traumas are also included in their etiology as a case reported as cornu cutaneum on the feet of the patient [6]. Our patient had a lesion in nasal vestibule supporting the traumatic etiological factors. He felt itching as the mass grew and he scratched; therefore, repeated traumas were made by the patient himself.

Cornu cutaneum is generally a slow developing mass. Our patient had nasal obstruction symptoms for more than 3 months and the mass became visible from outside the nose within a month. Cornu cutaneum is generally a benign lesion but is reported to have relation with malignant or premalignant diseases. Histological researches show that basal membrane invasion supporting differential diagnosis should include a wide range from seborrheic keratosis to squamous cell carcinoma [8, 9]. Predisposing factors include benign (seborrheic keratosis, viral verrucae, and molluscum contagiosum), premalignant (solar keratosis, arsenic keratosis, and Bowen's disease), and malignant (squamous cell carcinoma, basal cell carcinoma, metastatic renal cell carcinoma, granular cell tumours, sebaceous carcinomas, and Kaposi's sarcoma) diseases. Differential diagnosis can only be made by careful histopathological investigation of the basal lamina [1].

Excisional biopsy can be made for diagnosis and treatment. We treated and diagnosed our patient with excisional biopsy. Isolated cornu cutaneum has dead keratin cells without any living tissues [1]. Our pathological examination also showed papillomatous keratin lamellas without any alive cells.

As a result, as mostly seen in places which are sensitive to sunlight, cornu cutaneum from nasal vestibule is a rare disease [10] which is a slow developing benign lesion characterized by necrotic tissues without any living cells microscopically.

## 4. Conclusion

Apart from sunlight, traumas are included in etiology of cornu cutaneum. As mostly seen in places sensitive to sunlight, cornu cutaneum from nasal vestibule is uncommon suggesting the effect of traumas. Pathological examination also showed papillomatous keratin lamellas without any alive cells. There are a few reported cases of cornu cutaneum resulting from nasal vestibule. Our case is important as nasal vestibule is a rare place for cornu cutaneum to be seen.
